# Advances in therapeutic hybrid neuromodulation for Parkinson’s disease

**DOI:** 10.1007/s13205-025-04681-z

**Published:** 2026-01-05

**Authors:** Iqra Bano, Jaison Jeevanandam, Grygoriy Tsenov

**Affiliations:** 1 Faculty of Science, Department of Animal Physiology, Charles University, Albertov 6, Prague, 128 00 Czech Republic; 2 Division of Experimental Neurobiology, Preclinical Research Program, National Institute of Mental Health, Topolová 748, 250 67 Klecany, Czech Republic; 3 Faculty of Bioscience, Department of Physiology and Biochemistry, Shaheed Benazir Bhutto University of Veterinary and Animal Sciences, Sakrand, 67210 Pakistan

**Keywords:** Biotechnology, Parkinson’s disease, Focused ultrasound stimulation, Deep brain stimulation, Neuromodulation, Hybrid stimulation

## Abstract

Biotechnology is driving the next generation in neuromodulation therapies for Parkinson’s disease (PD), which is a progressive neurodegenerative disorder characterized by motor dysfunction due to the loss of dopaminergic neurons in the substantia nigra. While pharmacological therapies and Deep Brain Stimulation (DBS) are standard treatments, they often fail to fully address the non-motor impairments that significantly affect patients’ quality of life. A novel therapeutic strategy integrating Focused Ultrasound Stimulation (FUS) with DBS, known as hybrid stimulation, has emerged as a promising approach. This combined modality leverages the continuous neuromodulation of DBS with the non-invasive, precise targeting of FUS, enhancing therapeutic efficacy through complementary mechanisms. DBS modulates neural firing patterns and promotes neuroplasticity, while FUS allows for precise, transient disruption of the brain barrier (BBB), enhances drug delivery, and induces localized neuro-thermal effects, potentially aiding neuroprotection and neurotransmitter regulation. This review critically evaluates the role of DBS and FUS in PD treatment, focusing on the need for a hybrid DBS-FUS approach. We highlight emerging preclinical and clinical evidence of their synergistic effects in modulating dopamine synthesis, neurotransmitter dynamics, and synaptic remodeling. Furthermore, we present a computational bibliographic analysis to assess research trends, knowledge gaps, and the evolving impact of hybrid neuromodulation strategies, offering a comprehensive perspective on their potential to address both motor and non-motor symptoms of PD.

## Introduction

Parkinson’s Disease (PD) is a progressive neurodegenerative disorder that primarily affects movement, leading to debilitating symptoms such as tremors, bradykinesia, rigidity, and postural instability (Swinnen et al. 2025). According to a recent study examining data from 1980 to 2023, the global pooled prevalence of PD is 1.51 per 1000 cases, with a higher prevalence in males (1.54 per 1000 cases) compared to females (1.49 per 1000 cases) (Lin et al. 2021a). The prevalence of PD continues to rise with the aging global population, underscoring the urgent need for effective treatment strategies to manage the disease and modify its progression (Charvin et al. 2018; Singh and Rai 2025). Conventional biotechnological approaches, including pharmacological treatments, have provided symptomatic relief; however, they often fall short of addressing the disease’s progression and the long-term efficacy of symptom management (Goel et al. 2025; Jagadeesan et al. 2017). Recent progress in biotechnology and bioengineering has begun to transform neuromodulation therapies for neurodegenerative disorders (Singh et al. 2024; Kumar et al. 2022). Hybrid deep brain stimulation–focused ultrasound (DBS–FUS) exemplifies this convergence of advanced biomedical devices, molecular targeting, and computational intelligence (Bhattacharya et al. 2022). Yet, these advanced PD treatments face limitations due to their influence on individual cells, leading to alterations in network function and, ultimately, and eventually causing behavioral changes in patients (Rai and Singh 2020). DBS involves the implantation of electrodes into specific brain regions to deliver continuous electrical stimulation for modulating neuronal activity (Weaver et al. 2005). It is noteworthy that this technique can provide substantial symptomatic relief and improve the quality of life for numerous patients with advanced PD (Hariz and Blomstedt 2022). However, DBS also possesses certain limitations, including potential side effects and the need for precise electrode placement (Olson et al. 2023). On the other hand, FUS is a non-invasive neuromodulation technique that uses high-frequency sound waves to target and modulate specific brain regions (Lee et al. 2022). FUS offers the advantage of avoiding invasive procedures and can be precisely directed to the target area, potentially reducing the risk of adverse effects that are associated with invasive techniques (Bano et al. 2025). Still, FUS must be integrated with magnetic resonance imaging (MRI) to target specific brain regions, and errors in spatial precision of target location, as it is tedious to use ultrasound in the skull, are the limitations of this method (Liu et al. 2021). As a result, the concept of hybrid stimulation strategies, such as combining DBS and FUS, is gaining traction in neuromodulation research (Tian et al. 2023). Moreover, researchers have recently been aiming to harness the strengths of DBS and FUS modalities by integrating them, while mitigating their respective limitations (Rai et al. 2024; Yuksel et al. 2023). Some studies revealed that the synergy between DBS and FUS could potentially lead to enhanced therapeutic outcomes, which eventually offer highly effective and personalized treatment options for individuals with PD (Stefani et al. 2022). Lin et al. (2021) compared the efficacy of DBS and FUS in Parkinsonian tremors via a systematic review and network meta-analysis. The study revealed that the FUS integrated with MRI is not inferior to DBS in the intervention and suppression of Parkinsonian tremor (Lin et al. 2021a). While the research and development of the DBS-FUS combination for treating neurological diseases is still in its early stages, this review offers a comprehensive overview of conventional stimulation strategies in PD treatment, with a particular emphasis on the integration of DBS and FUS. This review critically evaluates the current evidence base, highlights emerging biotechnological directions, and provides a bibliometric perspective to identify opportunities and challenges in translating hybrid DBS–FUS into clinically actionable biotechnology-based therapies for Parkinson’s disease. To further enrich the analysis, we include a computational bibliographic study covering the period 2015–2025, offering quantitative insight into publication trends, research networks, and evolving scientific priorities in this rapidly developing field.

## Overview of common brain stimulation techniques

### Deep-Brain stimulations

**Fig. 1:**
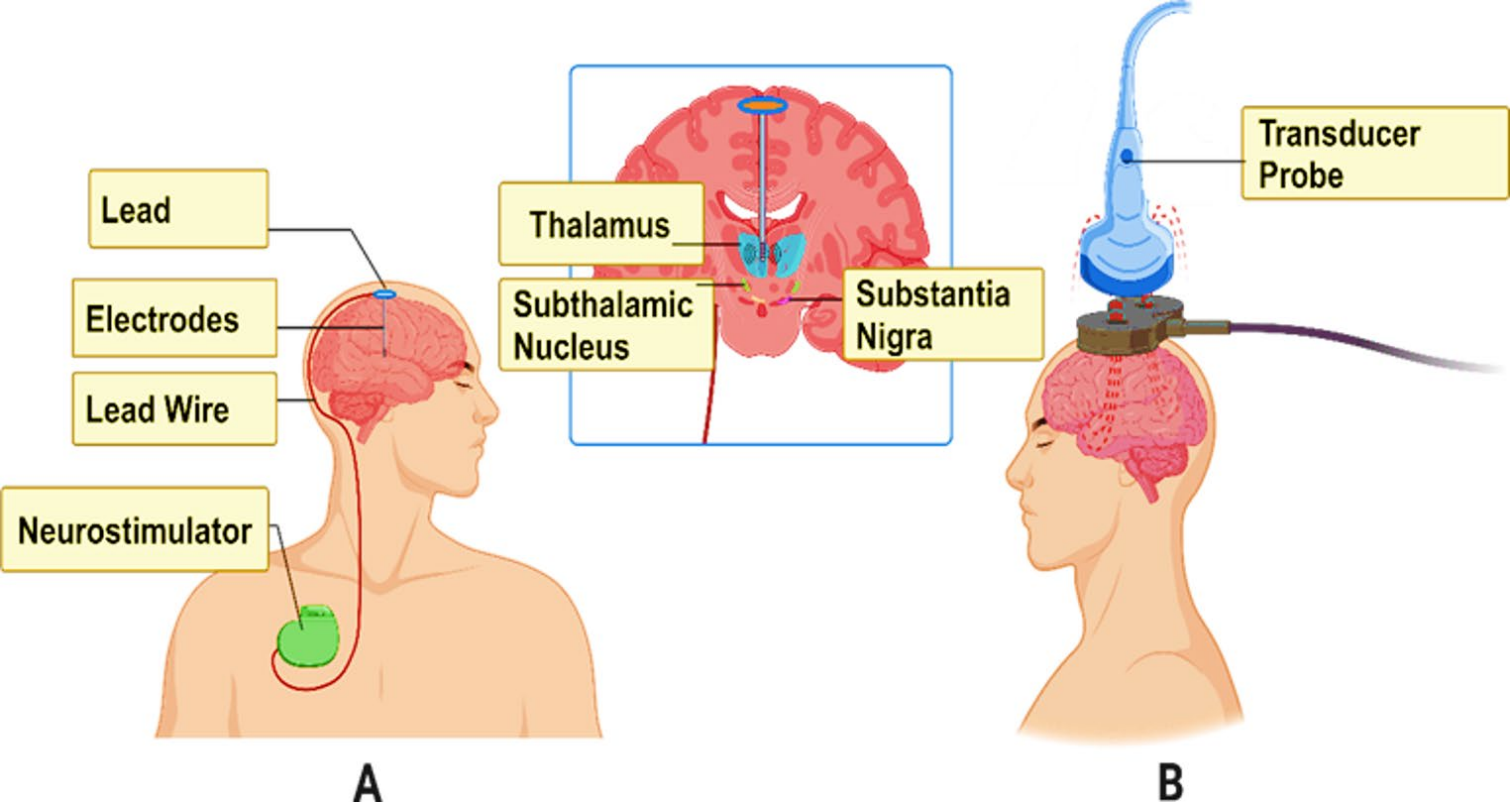
The image illustrates two advanced neuromodulation techniques used in PD treatment: **A** Deep Brain Stimulation (DBS) and **B** Focused Ultrasound (FUS). In panel (**A**), the DBS system is shown with implanted components, including electrodes inserted into the brain, typically targeting regions like the subthalamic nucleus, thalamus, or substantia nigra. The electrodes are connected via lead wires to a neurostimulator implanted in the chest, which delivers electrical impulses to regulate abnormal brain activity. In panel (**B**), the FUS system is depicted with a non-invasive transducer probe placed on the scalp. The probe emits FUS waves that can target deep brain structures to modulate neuronal activity without surgical implantation. Both techniques target similar brain regions implicated in motor control, offering complementary strategies for alleviating Parkinsonian symptoms through either electrical or acoustic stimulation

DBS operates by delivering continuous electrical stimulation to targeted brain regions, typically at frequencies between 100 and 160 Hz (Hariz and Blomstedt 2022). The electrodes are implanted into specific deep-brain nuclei, such as the subthalamic nucleus (STN), globus pallidus internus (GPi), or thalamus, depending on the condition being treated (Mahlknecht et al. 2022), as displayed in Fig. 1. These regions are involved in complex neural circuits that regulate motor function, emotion, and cognition. The precise mechanism of DBS in exhibiting therapeutic effects has not been completely reported yet; however, this approach can modulate pathological neural activity by several potential mechanisms (Chang et al. 2023). It is noteworthy that abnormal oscillatory activity within the basal ganglia circuitry is a key feature in disorders, such as PD (Hariz and Blomstedt 2022). Further, high-frequency stimulation through DBS can disrupt these pathological rhythms and eventually restore normal firing patterns of neurons. This disruption may prevent abnormal signal propagation and elevate symptoms (Liu et al. 2021). DBS possesses the ability to activate local inhibitory interneurons, which can suppress the activity of excitatory neurons within the targeted region. For instance, DBS can stimulate GABAergic neurons in the STN to inhibit excitatory output in the GPi and substantia nigra, which eventually reduces the excessive motor output characteristic of PD (França et al. 2022). Moreover, DBS has the potential to influence the release of various neurotransmitters, including dopamine, serotonin, and glutamate. Furthermore, DBS can alter the balance of excitation and contribute to inhibitory signals in the brain by modulating the release of neurotransmitter chemicals, which eventually contribute to its therapeutic effects (Davidson et al. 2024). It is noteworthy that abnormal oscillatory activity within the basal ganglia circuitry is a key feature in disorders, such as PD (França et al. 2022). Further, high-frequency stimulation through DBS can disrupt these pathological rhythms and eventually restore normal firing patterns of neurons (Sarica et al. 2023). This disruption may prevent the propagation of abnormal signals and elevate symptoms (Chang et al. 2023).

### Focused ultrasound stimulation

FUS aids in the treatment of PD by emitting acoustic waves that converge at a precise focal point within the brain (Singh and Reynolds 2024). The basic mechanism involves the use of ultrasound (US) transducer arrays to deliver mechanical energy to a small, targeted brain region (Krishna et al. 2018). The biological effects of FUS are governed by parameters such as frequency, intensity, and pulse duration of the ultrasound waves (Singh and Reynolds 2024). Initially, FUS exerts mechanical forces as ultrasound waves propagate through brain tissues (Baek et al. 2022). These forces can displace cell membranes, alter ion channel dynamics, and subsequently modulate neuronal excitability (McMahon et al. 2021). At higher intensities, FUS may induce cavitation, the formation and collapse of microbubbles in tissue, which, while typically associated with neuro-thermal damage, can be harnessed for controlled therapeutic applications such as transient disruption of the blood-brain barrier (BBB) to enhance drug delivery, and also produces localized heating effects (Chen et al. 2021). In the context of hybrid DBS–FUS, “neuro-thermal effects” refer strictly to low-intensity, non-ablative temperature elevations typically below 1–2 °C (Blackmore et al. 2023). These mild thermal shifts can enhance perfusion, neuronal responsiveness, and metabolic support but remain well within established neuromodulatory safety limits (Lin et al. 2021a). Importantly, the hybrid approach reviewed here does not employ ablative sonication; all discussed effects occur under sub-threshold thermal exposures that avoid tissue coagulation, cavitation damage, or irreversible heating (Bano et al. 2025). At low intensities, this thermal effect is minimal and non-damaging but sufficient to influence neural activity by affecting temperature-sensitive ion channels and synaptic enzymes (Bano et al. 2025). Additionally, FUS can increase cell membrane permeability through sonoporation, facilitating the entry of therapeutic agents or altering intracellular signaling cascades (McMahon et al. 2021). Consequently, FUS is increasingly being explored for cognitive enhancement, particularly for memory and attention improvement (Burgess and Hynynen 2013). Recent literature indicates that hybrid stimulation combining DBS and FUS can have synergistic effects on animal models by more effectively modulating PD-relevant neural circuits (Lin et al. 2021a). While DBS delivers continuous electrical stimulation to support motor function (Chang et al. 2023), FUS may be used to fine-tune this activity, potentially improving treatment outcomes and minimizing side effects (Bhattacharya et al. 2022). Furthermore, FUS can aid in targeted drug delivery across the BBB and promote neuroplasticity, enhancing the overall therapeutic impact of DBS (Meng et al. 2021). A comparative overview of DBS, FUS, and hybrid stimulation strategies for PD management is presented in Table 1.

**Table 1:** Comparative features of DBS, FUS, and hybrid stimulation strategies in PD treatment

Features	DBS	FUS	Hybrid stimulation strategies
Primary mechanism	Delivers continuous electrical pulses to modulate neuronal activity (Matt et al. 2024)	Uses high-intensity US waves to create localized lesions (Hynynen et al. 1996)	Combines DBS’s continuous stimulation with FUS’s non-invasive, precise targeting (Yoo et al. 2022)
Neurochemical changes	Alters neurotransmitter release, such as dopamine. Induces long-term changes in synaptic strength and neuronal structure (Davidson et al. 2024)	Low-intensity US influences neuronal activity via mechano-transduction. Temporarily enhances the delivery of therapeutic agents via disruption of the BBB (Burgess and Hynynen 2013)	Enhanced therapeutic outcomes for improved efficacy and reduced side effects (Lin et al. 2021a)
Targeting precision	Highly precise but invasive requires surgical implantation of electrodes and involves risks associated with invasion procedures (Mahlknecht et al. 2022)	FUS targets the specific brain regions with high accuracy. Performed without surgery, reducing procedural risks (Bano et al. 2025)	Integration of DBS and FUS can refine targeting precision and potentially reduce invasion. Non-complements the invasiveinvasive FUS nature of DBS (Bhattacharya et al. 2022)
Clinical applications	Effective for managing motor symptoms in advanced stages of PD. Common targets include the STN, GPi, and thalamus (Hariz and Blomstedt 2022)	Effective for reducing tremors, especially in essential tremors and PD. Neuromodulation for other symptoms, such as motor control and cognitive effects (Chen et al. 2021)	Hybrid approaches are still under investigation to improve treatment efficacy. Aiming to reduce side effects and personalized treatment (Lin et al. 2021a)
Advantages	Significant improvement in motor symptoms. Stimulation settings can be modified based on the patient’s response (Swinnen et al.)	Avoids surgical risks and complications. Allows for targeted treatment and adjustment. Minimizes procedural risks (Meng et al. 2021)	Potentially synergistic effects by combining the benefits of DBS and FUS for enhanced outcomes. Personalized treatment options through the ability to tailor approaches to individual needs (Lin et al. 2021b)
Challenges	Surgical risks include infection, bleeding, and cognitive effects. Possible side effects include mood changes and speech problems (Olson et al. 2023)	Requires exact targeting to avoid unintended effects. Limited long-term data and more studies are needed on long-term efficacy and safety (Singh and Reynolds 2024)	Combining DBS and FUS involves technical and logistical challenges. Requires coordination of two different stimulation methods. Need for thorough research to understand the combined effects (de Souza et al. 2025)

### FUS & BBB safety

Although FUS provides a powerful platform for neuromodulation and BBB modulation, its safety profile must be carefully considered when transitioning to hybrid DBS-FUS systems (Darrow 2019). Controlled BBB opening can unintentionally induce microvascular stress, leading to subtle endothelial injury, petechial microhemorrhages, or transient extravasation of serum proteins when acoustic pressures exceed recommended thresholds (Hsu et al. 2022; Burgess and Hynynen 2013). Crucially, the possibility of immune cells, lipophilic medications, or peripheral inflammatory mediators inadvertently entering the parenchyma is increased by the possibility that even well-regulated BBB opening may stay permeable for several hours (Jiang et al. 2025). Experimental and clinical imaging studies have reported localized neuroinflammatory signatures, microglial activation, cytokine upregulation, and perivascular macrophage recruitment following repeated BBB-modulating FUS sessions (Sinharay et al. 2019). It is also necessary to consider mechanical and thermal interactions (Lee et al. 2020b). Although low-intensity FUS typically generates very little heat, the buildup of acoustic energy close to calcified structures, scar tissue, or implanted devices may cause localized “hot spots” that might change neuronal excitability or encourage oedema (Collins and Mesce 2022). Cavitation dynamics, especially in the presence of microbubbles, must be tightly monitored since unstable cavitation can damage microvessels, disrupt tight junction integrity, and perturb ionic homeostasis (Xie et al. 2022). For hybrid DBS-FUS, additional device-related issues arise. Metallic components of DBS systems may subtly affect ultrasonic propagation, disperse acoustic energy, or change thermal deposition patterns (Winter et al. 2021). Therefore, safe clinical translation needs stringent adherence to acoustic dose limits, real-time cavitation monitoring, MRI-based thermometry, and established microbubble dosing procedures to assure consistent BBB opening and sustained neuromodulatory effects without structural compromises (Gupta et al. 2025).

## Therapeutic agents most suitable for FUS-Enabled delivery in PD

FUS-mediated BBB opening offers a unique opportunity to deliver pharmacological and biological agents directly to deep brain areas involved in PD (Baek et al. 2022). Several treatment classes have shown promising preclinical results when combined with ultrasound targeting (Alfihed et al. 2024). Neurotrophic factors such as glial cell line-derived neurotrophic factor (GDNF) and brain-derived neurotrophic factor (BDNF) promote dopaminergic neuron survival, enhance synaptic plasticity, and counteract striatal denervation, but have previously failed in clinical trials due to poor BBB penetration; FUS now allows for localized delivery directly to the putamen or substantia nigra (Ji et al. 2019). Antibody-based therapies, including anti-α-synuclein monoclonal antibodies and immunotherapies targeting oligomeric species, can be similarly directed into affected regions, offering a strategy for modifying pathogenic protein aggregation (Alfaidi et al. 2025). FUS-mediated BBB modulation allows gene therapy vectors, such as AAV2, AAV9, and lentiviral constructs, to deliver genes encoding tyrosine hydroxylase, aromatic L-amino acid decarboxylase (AADC), or enzymes supporting dopamine synthesis, as well as RNA interference constructs to reduce α-synuclein expression (Kofoed and Aubert 2024). Small-molecule medicines such as Nrf2 activators, mitochondrial protectants (CoQ10 derivatives, peptides), and anti-inflammatory drugs can reach therapeutic quantities within brain tissue when administered through transitory BBB openings (Dailah 2022). Collectively, these agents represent promising candidates for integration into hybrid DBS–FUS protocols, pairing network-level modulation with molecular therapies that target underlying neurodegenerative processes (Alfihed et al. 2024).

## Effects of hybrid DBS-FUS in PD models

PD presents a spectrum of motor and non-motor symptoms that significantly impair quality of life (Rai 2021; Rai et al. 2025). Motor symptoms include bradykinesia, resting tremors, dystonia, abnormal gait, and vocal changes, while non-motor manifestations involve cognitive decline, gastrointestinal dysfunction, excessive sweating, anosmia, and joint stiffness (Weintraub et al. 2022). Recent preclinical studies suggest that combining DBS with FUS yields superior therapeutic outcomes compared to DBS alone (Bloem et al. 2021). In rodent models of PD induced by 6-hydroxydopamine (6-OHDA), the addition of FUS to standard DBS protocols resulted in notable improvements in motor function, as evidenced by enhanced performance in behavioral tests such as the cylinder test, open field test, and apomorphine-induced rotation assessments (Wu et al. 2025). Animals receiving hybrid DBS-FUS therapy exhibited reduced rotational asymmetry and increased stride length, indicating better motor coordination and locomotor control (Lin et al. 2021b). These effects are thought to arise from FUS’s ability to modulate additional neural circuits and enhance the efficacy of DBS by promoting neuroplasticity and reorganizing dysfunctional brain networks (Wu et al. 2025).

### Mechanistic synergy between DBS and FUS

Hybrid DBS-FUS stimulation activates many complementary network-level pathways that jointly promote neuromodulation in PD (Lin et al. 2021a). DBS interrupts aberrant beta oscillations and inappropriate synchronization within the cortico-striatal-pallido-thalamo-cortical (CSPTC) loop, restoring more natural firing patterns in the STN and GPi and enhancing the fidelity of thalamocortical relay activity (Taghva et al. 2013). Recent research shows that DBS not only suppresses aberrant rhythms but also recruits compensatory gamma-band activity linked to enhanced motor start (Sirica et al. 2021). FUS enhances these effects by altering local circuit excitability through mechanical wave propagation (Collins and Mesce 2022). This changes membrane tension, adjusts the excitatory-inhibitory balance, and fine-tunes the response of neuronal ensembles (Ji et al. 2019). New in vivo work shows that low-intensity FUS can selectively modulate firing rates in the motor cortex and subthalamic circuits without altering surrounding structures, improving the precision of DBS-modulated pathways (Singh and Reynolds 2024; Leung et al. 2025). Furthermore, hybrid stimulation has been found to alleviate pathological thalamocortical dysrhythmia, a defining feature of PD, by improving phase resetting and spike-timing regularity (Liu et al. 2021). FUS’s ability to increase neurovascular coupling and cerebral blood flow further enhances DBS’s neuromodulatory depth by ensuring better metabolic support for high-frequency firing neurons (Alfihed et al. 2024). Network-connectivity analysis from recent animal EEG/LFP experiments shows that hybrid stimulation promotes greater desynchronisation of abnormal oscillations and more robust restoration of corticobasal ganglia coherence than DBS alone (He et al. 2025). Together, these circuit-level interactions create a more flexible, responsive, and stable neuromodulation environment, allowing for enhanced control of motor function and potentially reducing the required DBS current intensity, therefore minimizing stimulation-induced side effects (Lin et al. 2021c).

## Cellular & molecular crosstalk driving hybrid DBS–FUS synergy

At the microscopic level, hybrid DBS-FUS stimulation activates strong and convergent intracellular mechanisms that enhance neuroprotection, synaptic recovery, and long-term plasticity (Ferreira Felloni Borges et al. 2023). FUS activates mechanosensitive ion channels, including Piezo1/2, TRPV4, and TREK-1, causing fast Ca⁺ influx. This drives intracellular cascades such as CaMKII, ERK-MAPK, PI3K-Akt, and CREB pathways (Lee et al. 2020a). Recent research demonstrates that these pathways upregulate neurotrophic factors such as BDNF, GDNF, IGF-1, and NGF with significantly higher expression when FUS is paired with electrical neuromodulation (Zhu et al. 2023). DBS has several intracellular effects, including modifying vesicular dopamine release, lowering α-synuclein buildup, improving mitochondrial function, and restoring redox equilibrium through enhanced production of antioxidant enzymes, including SOD2 and catalase (Montgomery and Gale 2008). In theory, hybrid stimulation may amplify these benefits by aligning FUS-mediated Ca²⁺ signals with DBS-induced activity-dependent gene-expression programs, creating a “plasticity-permissive” intracellular state, though this mechanism has not yet been directly confirmed in preclinical models (França et al. 2022; Alfihed et al. 2024). Emerging neuromodulation research shows that FUS (alone) can increase dendritic-spine density, enhance synaptic-vesicle recycling, and elevate synaptic-protein expression (e.g., PSD-95, synaptophysin); however, these effects have not yet been demonstrated specifically after hybrid DBS–FUS stimulation (Hu et al. 2023).

## Non-Motor modulation by hybrid DBS–FUS

Beyond motor symptom improvement, the hybrid DBS–FUS approach shows strong theoretical and emerging experimental potential for alleviating non-motor symptoms, which originate from dysfunction in limbic, cortical, and autonomic circuits rather than the classical motor basal ganglia pathways (Chen et al. 2021). DBS alone can partially modulate these domains by influencing associative–limbic subdivisions of the STN and GPi and by shaping activity in the medial prefrontal cortex, anterior cingulate cortex, and ventral pallidum (Hariz and Blomstedt 2022). Recent preclinical and early human neuromodulation studies show that low-intensity FUS targeting fronto-limbic networks can reduce anxiety-like behavior, improve attentional control, and modulate resting-state connectivity (Davidson et al. 2025). Although these findings are not specific to PD, they suggest mechanisms that could complement DBS-driven stabilization of limbic circuitry (Cox et al. 2025). Hybrid stimulation may also benefit sleep disturbances, which are among the most disabling non-motor symptoms in PD (Ferreira Felloni Borges et al. 2023). DBS affects slow-wave and REM rhythms via projections to the thalamus and brainstem, whereas FUS can fine-tune thalamocortical oscillations and alter activity in the pedunculopontine nucleus and other arousal centers (Tseng et al. 2020; Bange et al. 2025). These data support hybrid DBS-FUS as a multimodal therapy capable of recalibrating limbic-cognitive networks, restoring sleep-wake physiology, and stabilizing autonomic function. Although there are no specific clinical trials on non-motor outcomes, growing research shows that hybrid stimulation has the potential to give broader and greater long-term improvements across the entire range of PD symptoms than DBS (Alfihed et al. 2024; França et al. 2022).

### Neuroprotective and electrophysiological evidence

Experimental findings show that animals treated with hybrid stimulation had reduced dopaminergic cell loss in the substantia nigra and better preservation of striatal dopamine levels (Lee et al. 2022). Notably, another researcher reported a decreased reduction in tyrosine hydroxylase-positive neurons in hybrid-stimulated rats following 6-OHDA lesioning, suggesting a potential to slow disease progression. Electrophysiological studies further support the benefits of hybrid neuromodulation (Gupta et al. 2025). Compared to DBS alone, hybrid stimulation more effectively modulates neural activity, altering local field potentials (LFPs) in the basal ganglia by reducing pathological beta-band oscillations and enhancing gamma-band activity associated with motor control. This neural rebalancing may allow clinicians to reduce DBS intensity, thereby minimizing side effects such as dysarthria or gait disturbances (Lin et al. 2021c). The combined approach also allows for precise lesioning of tremor-generating areas while maintaining continuous modulation of relevant circuits (Bloem et al. 2021). Enhanced motor coordination and reduced bradykinesia have been consistently reported in hybrid-stimulated animals, attributed to both functional modulation and potential neurorestorative effects (Levi et al. 2019).

### Clinical evidence supporting hybrid DBS–FUS

**Fig. 2:**
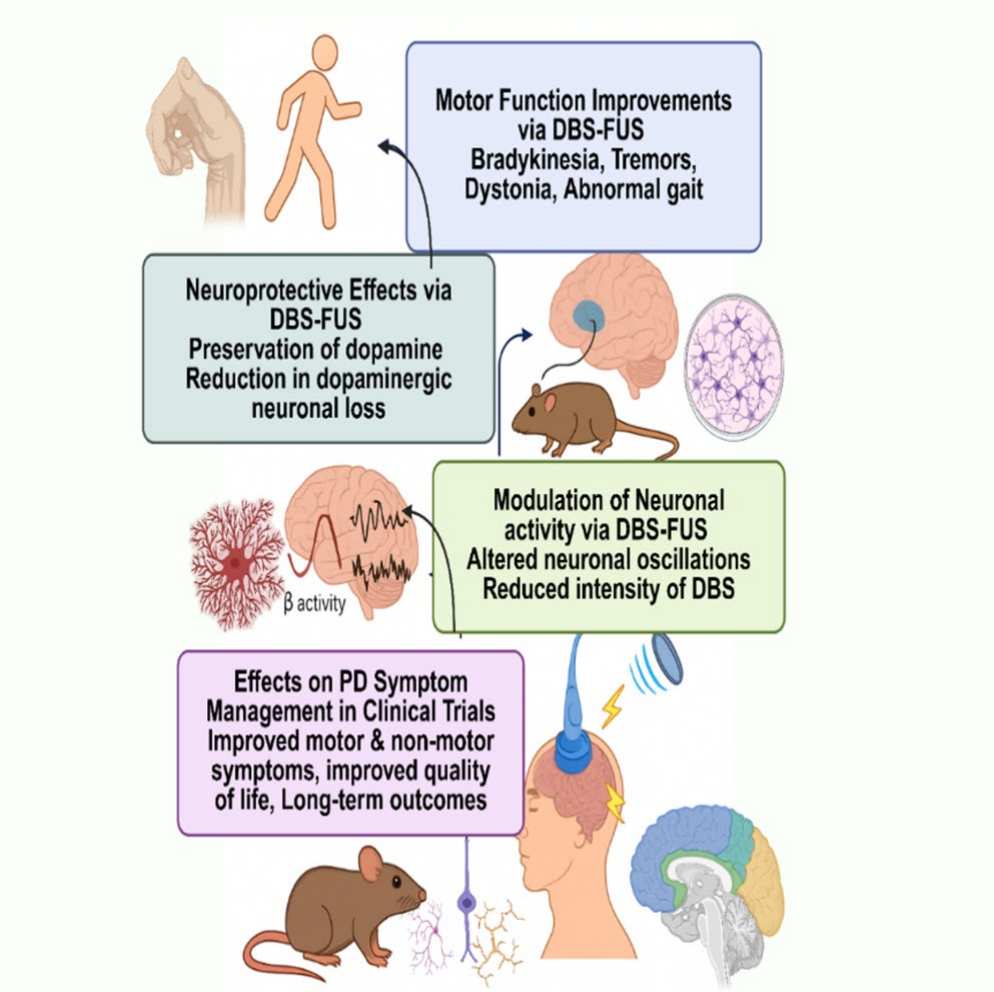
Illustration of the therapeutic effects of hybrid DBS-FUS in PD models. The image summarizes the key benefits of DBS-FUS, including improved motor function (bradykinesia, tremors, gait), neuroprotection through dopamine preservation, modulation of abnormal neuronal activity, and reduced DBS intensity. Clinical trials also show enhanced control of motor and non-motor symptoms, improved quality of life, and long-term outcomes

Clinical findings support the translational potential of this dual approach. While DBS effectively controls motor symptoms like tremors and rigidity, it has a limited impact on non-motor symptoms (França et al. 2022). Integrating FUS into the treatment plan allows for targeted modulation of the BBB, extending benefits to mood regulation, cognitive function, and autonomic balance (Fig. 2). Studies have shown that this synergistic strategy can offer real-time adjustability and a holistic therapeutic effect (Bhattacharya et al. 2022). Martinez-Fernandez et al. (2018) demonstrated that unilateral FUS thalamotomy significantly improved tremors and motor function in medication-refractory PD patients, with sustained effects and minimal adverse events at one-year follow-up (Martínez-Fernández et al. 2021). Similarly, another researcher reported that patients undergoing combined DBS-FUS therapy experienced greater improvements in both motor and non-motor domains compared to those treated with DBS alone. Additional multicenter trials confirmed the efficacy of FUS in reducing tremors and improving daily function, with benefits lasting up to 24 months (Kim et al. 2018). Altogether, these findings emphasize the promise of hybrid DBS-FUS neuromodulation as a transformative approach to PD therapy, potentially enabling personalized, dynamic, and comprehensive management of both motor and non-motor symptoms (Liang et al. 2025).

### Bibliometric analysis of DBS and FUS research (2015–2025)

**Fig. 3:**
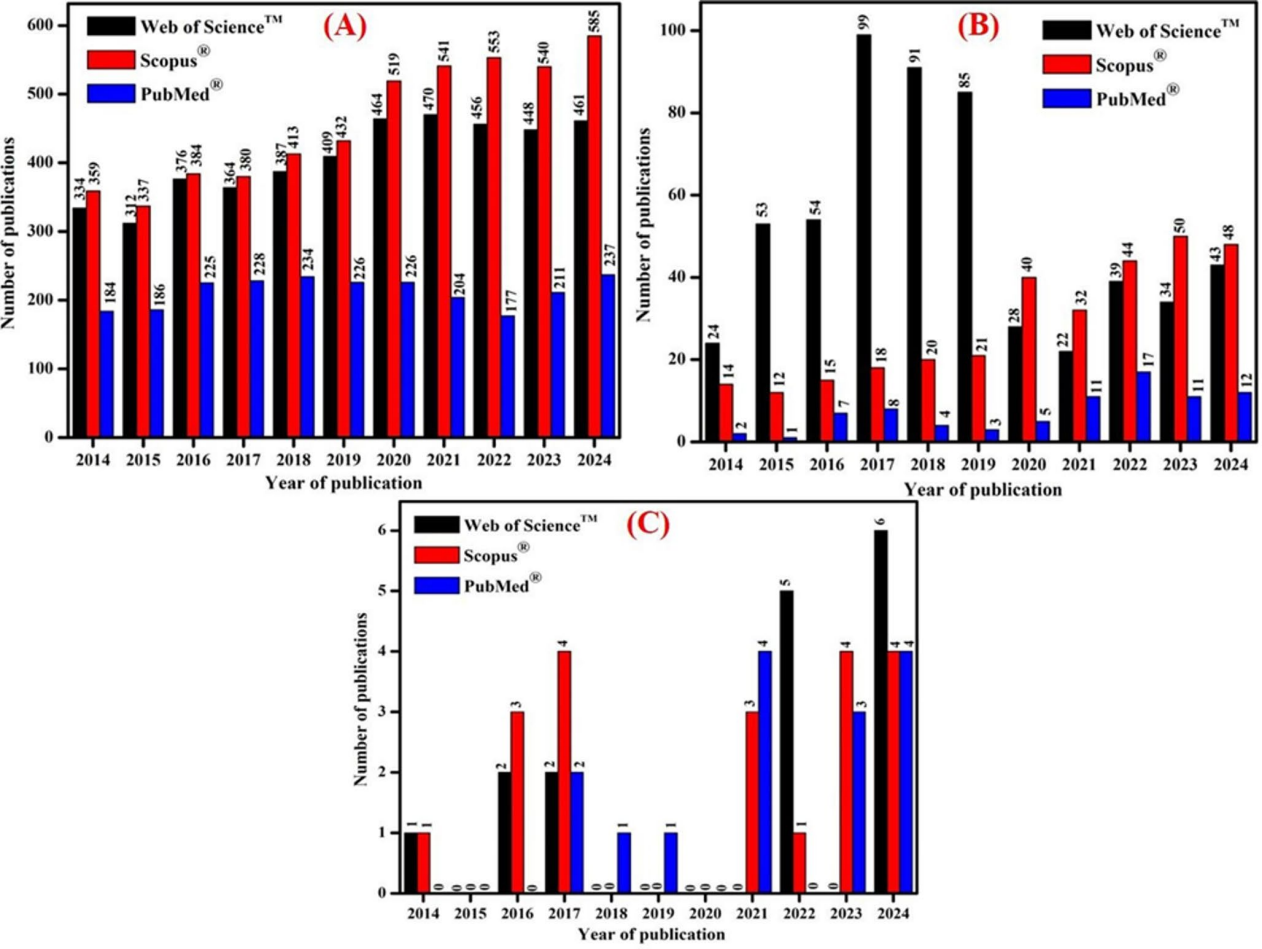
Number of publications for the past ten years with the keywords: **A** (“Parkinson’s disease” OR “PD”) AND (“Deep brain stimulation” OR “DBS”); **B** (“Parkinson’s disease” OR “PD”) AND (“Focused ultrasound stimulation” or “FUS”); and **C** (“Parkinson’s disease” OR “PD”) ”) AND (“Deep brain stimulation” OR “DBS”) AND (“Focused ultrasound stimulation” or “FUS”)

**Fig. 4:**
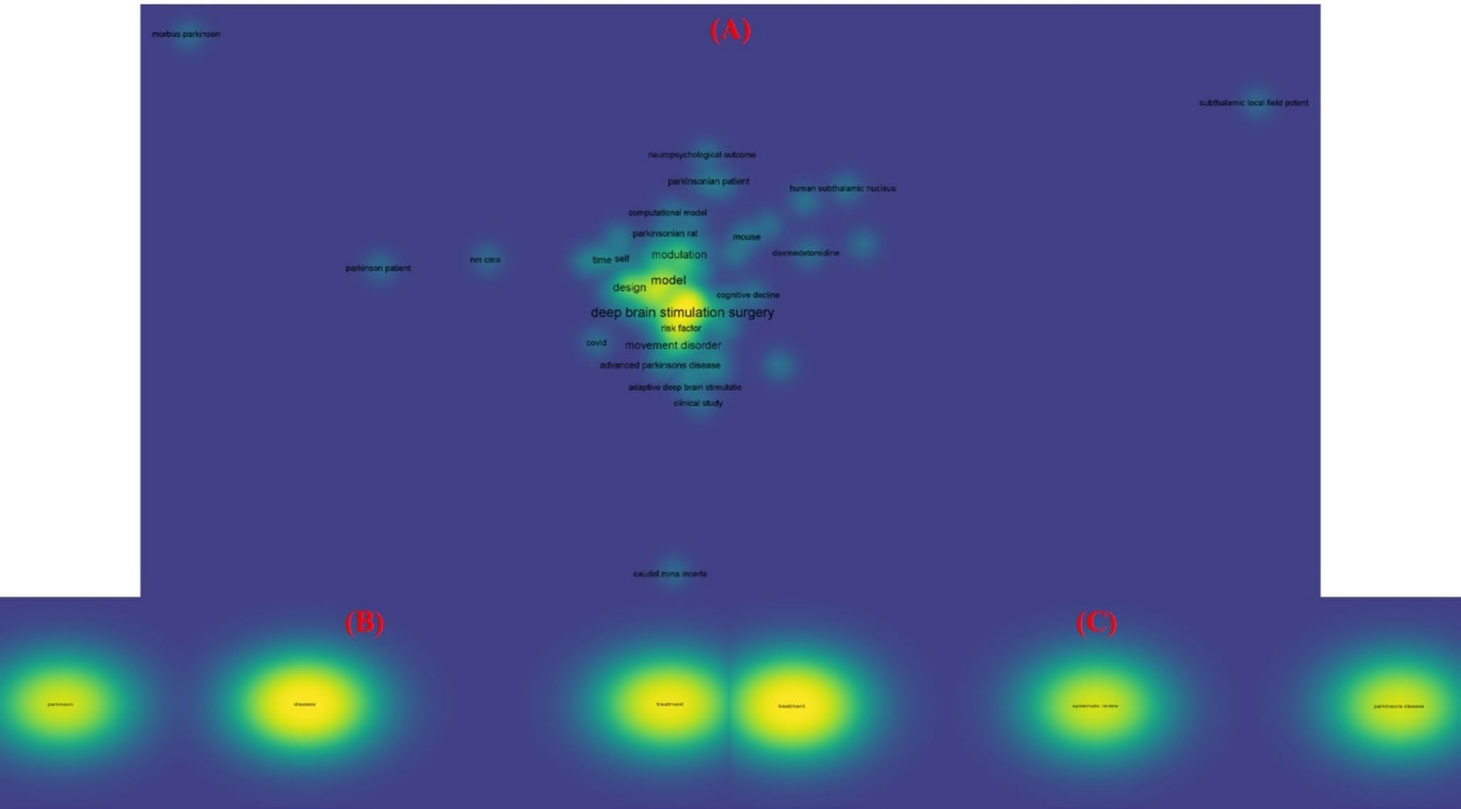
Network visualization maps of publication analysis using Web of ScienceTM, Scopus^®^ and PubMed^®^ for the keywords **A** (“Parkinson’s disease” OR “PD”) AND (“Deep brain stimulation” OR “DBS”); **B** (“Parkinson’s disease” OR “PD”) AND (“Focused ultrasound stimulation” or “FUS”); and **C** (“Parkinson’s disease” OR “PD”) ”) AND (“Deep brain stimulation” OR “DBS”) AND (“Focused ultrasound stimulation” or “FUS”). Note: The images are of high quality and zoomed in (250%) to view the links, terms, and networks

To increase clarity and repeatability, the bibliometric workflow was modified and organised using conventional scientometric techniques. Publications were retrieved following a multi-step strategy consisting of (i) keyword-based searching, (ii) database-specific filtering, and (iii) manual screening for relevance. Searches were performed in Web of Science™, Scopus^®^, and PubMed^®^ using Boolean combinations of “Parkinson’s disease/PD,” “Deep brain stimulation/DBS,” “Focused ultrasound/FUS,” and “DBS–FUS,” covering the period 2014–2024. Only peer-reviewed full-length publications were included, whereas conference papers, book chapters, retracted articles, preprints, brief communications, editorials, and comments were removed to guarantee analytical consistency across databases. After retrieval, duplicates were removed, and the remaining articles were screened based on titles, abstracts, and keywords to confirm relevance to PD neuromodulation. The inclusion criteria were: (i) experimental, clinical, or review studies specifically addressing DBS, FUS, or hybrid DBS–FUS in PD; (ii) articles reporting biological, mechanical, clinical, or technological outcomes; and (iii) studies related to neuromodulation mechanisms or therapeutic applications. Articles focusing solely on other neurological conditions without PD relevance were excluded. For analysis, bibliographic data, including titles, authors, keywords, abstracts, citations, and publishing journals, were exported into VOSviewer (version 1.6.19). Keyword co-occurrence mapping was applied to identify dominant research clusters, emerging themes, and under-explored areas. Cluster density maps were used to visualize the intensity of thematic research, while link-strength scores quantified the relationship between DBS and FUS research communities (Figs. 3 and 4).

The analysis disclosed three major trends:

1. DBS dominates the PD neuromodulation literature, forming a dense, high-connectivity cluster reflecting its long-standing clinical use.
2. FUS research has undergone rapid expansion since 2019, driven by advances in MRI-guided targeting and BBB-opening technologies.
3. Hybrid DBS–FUS forms the smallest but fastest-growing cluster, indicating that combined neuromodulation remains in its early development phase but is gaining significant attention, particularly in the last 2–3 years.

The bibliometric analysis also identifies some significant knowledge gaps that are underexplored in current neuromodulation research. First, the co-occurrence patterns demonstrate that DBS and FUS constitute essentially distinct study clusters with little overlap, implying that mechanistic, engineering, and translational studies combining both modalities are limited. Second, publications focusing on hybrid DBS-FUS for PD make up the smallest cluster, confirming that most of the available literature regards both technologies as distinct rather than complementary therapies. Third, the VOSviewer mapping finds a small number of papers on non-motor symptoms, closed-loop control, and personalized stimulation biomarkers, all of which are critical components of next-generation neuromodulation. Finally, the bibliographic network reveals an under-representation of research involving patient stratification, BBB-enabled therapeutic delivery, and long-term safety of repeated ultrasound exposure in implanted DBS users. Together, these gaps emphasize the need for targeted interdisciplinary studies to establish the mechanistic foundations, safety parameters, and clinical protocols required to advance hybrid DBS–FUS toward practical therapeutic implementation.

### New directions in hybrid DBS-FUS research for novel PD treatment applications

#### Biotech-Enabled therapeutic delivery in hybrid DBS–FUS systems

Recent advancements in biotechnology have positioned hybrid DBS-FUS devices as revolutionary technologies for the precision delivery of next-generation medicines. According to recent research, FUS-enhanced delivery facilitates the transfer of AAV variants with greater tropism for dopaminergic neurons, CRISPR-based gene-editing systems that target α-synuclein regulatory pathways, and designer peptides that boost mitochondrial stabilization or synaptic repair (Gao 2024; Wang and Hegde 2020). Recent improvements in transcranial focused ultrasound (tFUS), which does not need microbubbles or BBB opening, show that low-intensity ultrasound may influence cortical and subcortical circuits with millimeter accuracy (Lu et al. 2021). Recent studies revealed that tFUS increases cognitive flexibility, lowers anxiety-like behavior, and modulates thalamocortical oscillations, indicating that ultrasound alone may consistently adjust neuronal excitability and network connection processes that complement those targeted by DBS (Shi et al. 2025). Adaptive DBS (aDBS) also provides an important foundation: its use of real-time beta-band feedback, circuit-state targeting, and oscillation-specific stimulation has produced superior motor outcomes and fewer side effects compared with continuous DBS (Stanslaski et al. 2024). Other non-invasive stimulation techniques strengthen this multimodal framework. Transcranial magnetic stimulation (TMS) and transcranial alternating current stimulation (tACS) demonstrate that combining electrical or magnetic stimulation with frequency-tuned modulation can create synergistic and circuit-selective effects (Shpektor et al. 2017). In parallel, biotechnology innovations such as BBB-permeabilizing nanoparticles, stimuli-responsive drug carriers, and biodegradable polymeric vesicles are now being integrated with FUS protocols to achieve temporal control of release profiles within DBS-responsive regions (McBenedict et al. 2024). Furthermore, FUS-enabled drug delivery has a distinct advantage over adaptive DBS and intraparenchymal drug delivery devices, according to recent biotechnological research (Alfihed et al. 2024). FUS can non-invasively transfer large-molecule treatments (neurotrophic factors, antibodies, viral vectors) over the BBB, in contrast to aDBS, which modifies electrical rhythms but is unable to transport biologics (Stanslaski et al. 2024). FUS accomplishes spatially limited, reproducible BBB opening without tissue penetration, in contrast to intraparenchymal injections, which need open surgery and have limited diffusion (Alfihed et al. 2024). This establishes hybrid DBS-FUS as a platform for biotech-enabled precision treatments, such as gene therapies, RNA interference complexes, programmable nanoparticles, and disease-modifying biologics that are unable to enter the brain by traditional means, in addition to neuromodulation (Lin et al. 2021a).

### Personalized stimulation protocols

Advanced neuroimaging techniques, such as functional magnetic resonance imaging (fMRI), positron emission tomography (PET) scans, and a high-definition electrophysiological mapping approach, can identify the specific brain networks disrupted by individual patients with PD (Ni 2023). Hence, it is possible to personalize stimulation parameters, which are tailored to each patient’s unique brain activity patterns by integrating the imaging modalities with DBS and FUS (Charvin et al. 2018). Furthermore, including genetic, molecular, and metabolic biomarkers could significantly enhance the personalization of DBS and FUS therapies. For example, certain genetic profiles can predict a better response to a hybrid DBS-FUS approach, while other patients might highly benefit from either DBS or FUS modality (Burgess and Hynynen 2013).

### Exploring hybrid DBS-FUS for sleep disturbance in PD

**Fig. 5:**
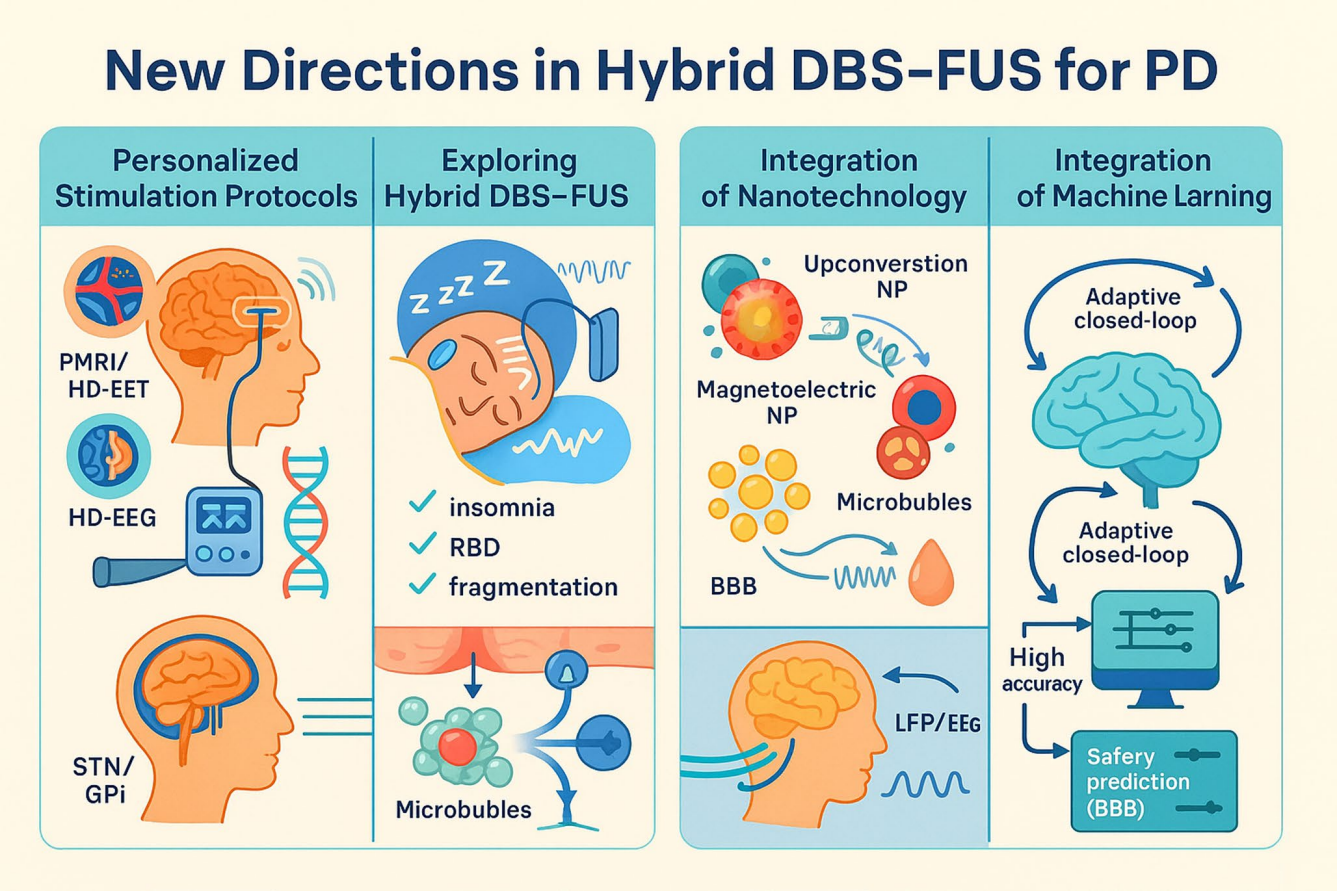
New directions in hybrid DBS–FUS research for PD, including approaches for personalized stimulation protocols integrating advanced neuroimaging and biomarkers; sleep-focused DBS–FUS therapy to alleviate PD-related sleep disturbances; nanotechnology integration to enhance targeted neuromodulation and drug delivery; and ML–driven adaptive systems for real-time optimization of stimulation and feedback. Abbreviations: PET (positron emission tomography), HD-EEG (high-density electroencephalography), LFP (local field potential), NPs (nanoparticle), RBD (rapid eye movement sleep disorder), STN (subthalamic nucleus), GPi (globus pallidus internus)

DBS has been identified to influence sleep-wake cycles in PD patients (Fig. 5), providing valuable insights into its potential to address sleep-related disturbances (Santyr et al. 2025). Generally, PD patients experience significant sleep disturbances, including insomnia, rapid eye movement (REM), sleep behavior disorder (RBD), excessive daytime sleepiness, and fragmented sleep (Lajoie et al. 2021). These issues can greatly impact patients’ quality of life and are often poorly addressed by conventional therapy (Thangaleela et al. 2023). Studies have demonstrated that DBS, particularly when targeting the STN or GPi, can benefit sleep in PD patients. It is noteworthy that DBS can improve sleep quality by restoring normal brain network activity and neurotransmitter balance (Chang et al. 2023). In certain cases, patients undergoing DBS have reported improved sleep duration and reduced sleep fragmentation, which are key components of sleep disorders in PD (Bloem et al. 2021). Additionally, DBS appears to reduce the frequency of nighttime awakenings, leading to restful and restorative sleep (Chang et al. 2023). Moreover, overstimulation or incorrect stimulation parameters can exacerbate sleep disturbances or lead to side effects such as insomnia or vivid dreams in certain cases (Sharma et al. 2018). Therefore, precise adjustment of stimulation parameters, as well as patient-specific personalization of the DBS treatment, is essential to optimize the therapeutic effects on sleep in PD (França et al. 2022).The mechanistic pathways outlined in this manuscript suggest a plausible framework for modulating limbic and cortical circuits; however, there is currently no published preclinical or clinical evidence directly demonstrating improvements in PD non-motor symptoms under hybrid DBS–FUS protocols. Therefore, the non-motor benefits discussed remain theoretical and warrant dedicated future investigation.

### Integration of nanotechnology in hybrid DBS-FUS

Recently, the integration of nanotechnology has been gaining significant attention among researchers to implement it in DBS and FUS due to its high surface-to-volume ratio and surface-dependent properties (Liu et al. 2025). Sun et al. (2023) reported that photothermal and upconversion hybrid nanoparticles are highly beneficial for tether-free DBS. In this study, silica-coated upconversion nanoparticles with the chemical composition of NaYF4:Yb/Er (Sodium-Yttrium-tetrafluoride: Ytterbium/Erbium) and NaYF4:Yb/Tm (Sodium-Yttrium-tetrafluoride: Ytterbium/Thulium) have been synthesized and are identified to be able to modulate neuronal activities via thermo- and photo-stimulation. The results revealed that the stereotactically injected nanoparticles in the mouse model possess bidirectional deep-brain modulation of feeding behavior, which can be achieved under tether-free illumination of 980–808 nm in the ChR2-expressing lateral hypothalamus region (Sun et al. 2023). Similar magneto-electric nanoparticles were fabricated by Nguyen et al. (2021) for in vivo wireless DBS. In this study, a magnetic field was used to activate and guide the nanoparticles toward the targeted brain region for brain activity stimulation. The results showed that the nanoparticles possess the ability to evoke rapid neuronal responses in cortical slices of the mouse model without a significant increase in the astrocyte and microglia count. Similar to DBS, nanoparticles have been under extensive research in FUS, especially targeting the brain (Nguyen et al. 2021). Likewise, another researcher listed several advancements in the usage of FUS for the delivery of magnetic resonance image-guided therapeutic nanoparticles to the central nervous system. The article emphasized that FUS and microbubbles are highly useful for regional targeting in the brain; however face challenges in penetrating the mesh-like, electrostatically charged brain parenchyma (Rouhi et al. 2024). Hence, nanoparticles integrated with FUS can be used to overcome the limitations of conventional FUS, due to enhanced targeted cellular uptake and controlled drug liberation, distribution, and excretion rate (Liu et al. 2025).

### Integration of machine learning in hybrid DBS-FUS

Recent advancements suggest that the integration of machine learning (ML) techniques into hybrid DBS-FUS frameworks may significantly enhance treatment outcomes in PD. ML-guided electrode systems, such as those employing lead-free piezoelectric nanoparticles, have demonstrated high diagnostic accuracy, achieving 99.1% sensitivity and 98.2% specificity in patient-specific DBS targeting and modulation (Eid et al. 2024). Adaptive DBS systems incorporating ML-based signal decoding algorithms have further enabled real-time optimization of stimulation parameters. These approaches utilize complex classification and regression models, supporting intelligent adjustments based on evolving neural dynamics and providing a platform for personalized, responsive neuromodulation (Oliveira et al. 2023). In the context of FUS, ML has been employed to predict and monitor BBB opening with high accuracy. One approach combined acoustic feedback-controlled nanobubble-assisted FUS (f₀ = 0.5 MHz) with a modified support vector data description (mSVDD) model. This system yielded a BBB opening efficacy of 85 ± 16.6% and a safety prediction rate of 62.5 ± 12.8% (Li et al. 2023). A complementary study utilizing a transformer-based multiple-instance learning model reported 96.7% prediction accuracy in rodent models, highlighting the potential of deep learning frameworks for BBB modulation (Dai et al. 2025). Collectively, these findings indicate that ML-driven approaches are likely to play a critical role in the future development of individualized and dynamically adjustable neuromodulation therapies for PD and related disorders (Twala 2025).

### Technical and clinical limitations of hybrid DBS–FUS systems

Hybrid DBS–FUS neuromodulation offers promising therapeutic potential, yet several limitations and adverse effects require careful consideration (Ferreira Felloni Borges et al. 2023). While human studies provide early clinical relevance, preclinical models remain essential for understanding tissue-level responses and optimizing stimulation parameters (Nair and Weiskirchen 2025). Successful implementation depends on careful patient selection, precise targeting, and continuous monitoring, as cavitation and thermal-related risks are central concerns in FUS-based therapies (Ferreira Felloni Borges et al. 2023). Moreover, the heterogeneity of PD progression and anatomical variability across patients underscores the need for personalized approaches (Harary et al. 2019). Despite advances, long-term safety, overstimulation risks, and durability of combined stimulation remain underexplored. The major technical and clinical limitations are outlined below.

### Engineering and Device-Integration challenges

A key engineering challenge is still synchronizing pulsed acoustic stimulation from FUS with continuous high-frequency electrical stimulation from DBS. Coordinated hybrid administration is technically challenging, especially for closed-loop or state-dependent neuromodulation, because these modalities have different duty cycles, temporal dynamics, and action mechanisms (Dallapiazza et al. 2015). Integration is made more difficult by metallic DBS electrodes, which can reflect, scatter, or distort ultrasonic waves, causing erratic changes in focal energy or localized micro-heating (Pichardo 2023). Although the modelling studies have not yet quantified the effect of DBS leads on FUS focal pressure, work on skull and cranial heterogeneity demonstrates large variability in focal intensity (~ 30–40% uncertainty), and ex vivo studies with DBS devices raise concerns regarding targeting shifts and implant-associated heating (Li et al. 2025).

### PD heterogeneity and the need for patient stratification

There is significant clinical and biochemical variation in PD, including tremor-dominant, akinetic-rigid, and PIGD phenotypes, each of which involves unique network dysfunctions (Deng et al. 2025). Both DBS and FUS responsiveness are affected by these variations. Additionally, myelination, skull anatomy, and regional microvascular density all affect US sensitivity (Li et al. 2024; França et al. 2022). Therefore, biomarker-driven stratification could be necessary for hybrid neuromodulation (Rektorová et al. 2025). Imaging indicators (neuromelanin-MRI integrity, DTI connectivity loss, PET dopaminergic impairments), neurophysiological markers (beta-band load, gamma enhancement, thalamocortical dysrhythmia), and genetic profiles (LRRK2, GBA) can all be used to predict treatment success (Zhang et al. 2015). Successful clinical translation requires customizing stimulation strength, acoustic dosage, pulse patterns, and timing to each patient’s neurobiological profile, which is consistent with precision-medicine methodologies (Little et al. 2013).

### Thermal and material interactions with implanted DBS electrodes

The interaction of FUS with implanted DBS leads presents a unique safety challenge (Dallapiazza et al. 2015). FUS may be reflected or scattered by the metallic contacts and conductive elements of electrodes, which have acoustic characteristics different from those of brain tissue (Antoniou et al. 2023). These interactions might result in thermal “hot spots” surrounding the electrode shaft, localized micro heating, or uneven energy deposition (Wessapan and Rattanadecho 2024). Glial encapsulation, tissue impedance, and current distribution at the electrode–tissue interface can all be somewhat changed by even low-intensity FUS exposures (Salatino et al. 2017). Concerns regarding long-term durability may arise from the accumulated mechanical or thermal stress that repeated FUS sessions may place on insulating materials and connections. To determine safe values, thorough temperature simulations and material-compatibility evaluations are required (Blackmore et al. 2023).

### Clinical translation and regulatory barriers

The lack of standardized monitoring instruments for hybrid treatment, the reliance on MRI, and the requirement for technical personnel with acoustic calibration skills complicate the clinical process (Martínez-Fernández et al. 2021). Patients with microvascular pathology, cognitive decline, or atypical PD may be more vulnerable to recurrent BBB openings, and patient selection criteria are yet to be well established (Sigona and Caskey 2024). As hybrid DBS-FUS techniques progress towards clinical application, coordinated engineering, neurology, neurosurgery, and industry collaboration will be necessary to overcome these regulatory, operational, and patient-specific obstacles (Lin et al. 2021a).

### Anatomical variability and its impact on treatment precision

Ultrasound transmission and focusing accuracy are greatly impacted by patient-specific characteristics, such as skull thickness, curvature, bone density, and asymmetry (Kong et al. 2021). Individual disparities in acoustic dosages may result from these anatomical variations. Chronic tissue alterations, such as gliosis, changed impedance, and microvascular remodeling, can affect how some areas react to FUS in individuals who have already had DBS leads implanted (Krokhmal et al. 2025). To preserve accuracy and safety, this variability makes standardized treatment more difficult and calls for customized acoustic modelling (Gupta et al. 2025).

### Long-term safety, tissue Integrity, and biological risks

The long-term consequences of combining repeated BBB opening with chronic electrical stimulation remain insufficiently defined (Radjenovic et al. 2022). Concerns include cumulative neuroinflammation, microvascular injury, delayed permeability changes, microhemorrhages, glial activation, and alterations in the electrode tissue interface (Lee et al. 2020b). Electrode heating during FUS exposures and potential overstimulation effects raise additional safety issues. These unknowns highlight the importance of longitudinal human studies and advanced in vivo monitoring tools to define safe cumulative exposure thresholds (Radjenovic et al. 2022).

## Conclusion and future directions

Hybrid DBS-FUS stimulation shows great promise for revolutionizing PD treatment by offering a holistic, personalized, and effective therapeutic approach. The integration of advanced neuroimaging, biomarker-guided treatment protocols, and closed-loop systems will be instrumental in optimizing and individualizing treatment regimens due to the progression of the field. Future research will be crucial in the determination of the best approach to incorporate hybrid stimulation into clinical practice to provide long-term relief and neuroprotection to PD patients worldwide. As the hybrid DBS–FUS technology advances toward clinical translation, several ethical, regulatory, and economic considerations must be addressed. The integration of an implanted neuromodulation system with an external ultrasound platform raises questions regarding long-term device safety, implant durability, and cumulative exposure to repeated FUS sessions, areas where long-term human data are lacking. Ethical concerns also arise from the possibility of unintended neuropsychiatric effects, especially when targeting limbic or cognitive circuits. Patient acceptability is another critical factor, as hybrid stimulation requires repeated imaging, technical calibration, and potential BBB-opening procedures that may increase treatment burden. Regulatory approval pathways are complex: DBS systems are classified as implantable Class III medical devices, while FUS platforms fall under separate imaging and therapeutic categories. A combined system will require harmonized safety standards, rigorous preclinical validation, and compatibility testing to ensure that acoustic energy does not interfere with implanted hardware. Cost considerations further shape the feasibility of hybrid approaches, as the combined use of MRI-guided FUS, implanted electrodes, and long-term programming may limit accessibility in many healthcare settings. Addressing these ethical, regulatory, and economic dimensions is essential to ensure responsible development and equitable adoption of hybrid neuromodulation therapies. Optimizing combination stimulation parameters in preclinical models, finishing long-term safety studies incorporating implanted hardware, and creating trustworthy biomarkers and imaging techniques to direct customized hybrid stimulation will all be necessary for future advancements. Additionally, FUS-enabled delivery of biologics, neurotrophic factors, antibodies, and gene therapies directly into deep brain structures must be validated. Additionally, early-phase clinical trials assessing patient selection criteria, therapeutic effect durability, and feasibility must be initiated. When taken as a whole, these research avenues will help hybrid DBS-FUS develop into a dependable, customized, and therapeutically revolutionary treatment that can address both the motor and non-motor aspects of PD. To further strengthen translational readiness, future research should also include (i) systematic testing of hybrid DBS–FUS in established PD animal models to evaluate motor and non-motor outcomes, (ii) mapping hybrid stimulation effects on limbic, cognitive, and sleep-related circuits using in vivo electrophysiology and functional imaging, (iii) developing standardized sonication–stimulation timing protocols, (iv) engineering real-time monitoring tools to track cavitation, thermal deposition, and electrode–ultrasound interactions, and (v) defining patient stratification frameworks based on imaging biomarkers, genetic profiles, or disease stage. Collectively, these efforts will establish the mechanistic foundations, safety thresholds, and clinical workflows required to move hybrid DBS–FUS toward first-in-human feasibility trials and, ultimately, scalable clinical application.

## Data Availability

Additional resources shall be provided upon inquiry to the corresponding author.
